# Outcome of medical and surgical management in intractable idiopathic trigeminal neuralgia

**DOI:** 10.4103/0972-2327.56317

**Published:** 2009

**Authors:** Hassan Salama, Hesham Ben-Khayal, Mohamed Abdel Salam Mohamed, Ashraf El-Mitwalli, Ashraf Ahmed Zaher, Ashraf EzzEldin, Hatem Badr, Peter Vorkapic

**Affiliations:** Department of Neurology and Neurosurgery, Sebea Hospital, Tripoli, Libya; 2 Department of Neurology Mansoura University, Egypt; 3 Department of Neurosurgery, Mansoura University, Germany; 4 Department of Neurosurgery, Hanover University, Germany,

**Keywords:** Microvascular decompression, trigeminal neuralgia

## Abstract

**Background:**

The neurovascular conflict in trigeminal neuralgia is an intractable condition; medical treatment is usually of long duration and can be annoying for both patients and clinicians.

**Aim:**

This prospective study was designed to assess the outcome of microvascular decompression (MVD) in patients with more than 3 years' history of intractable idiopathic trigeminal neuralgia (TN) and poor response to drugs.

**Materials and Methods:**

Twenty-one patients (8 females and 13 males) with intractable idiopathic TN (group 1) underwent MVD and were followed up for 2 years. Group 2 (n = 15), which included 6 females and 9 males, received pharmacotherapy. The outcome responses of pain relief were evaluated using a 10-cm visual analog scale (VAS) and the Barrow Neurological Institute (BNI) scoring system. The patients' morbidity was recorded as well.

**Results:**

All patients fulfilling the inclusion criteria were offered MVD surgery. Freedom from pain was achieved immediately after surgery in 95.2% (n = 20) of patients in group 1, and 90.5% (n = 19) had sustained relief over the follow-up period. There were no statistical significance recurrences or surgical complications in group 1 (*P*>0.5), while 53.3% (n = 8) of the subjects in group 2 showed poor response with pharmacotherapy over the same period of time and many patients experienced drug intolerance that had statistical significance (*P*<0.01).

**Conclusion:**

Early MVD in TN can help patients avoid the side effects of drugs and the adverse psychological effects of long-term pharmacotherapy and prolonged morbidity.

## Introduction

Idiopathic trigeminal neuralgia (TN), or tic douloureux, is a painful condition affecting the face. It is commonly unilateral and is characterized by brief attacks of lancinating neuropathic pain over the face that lasts from several seconds up to 2 min; it may either be provoked or may occur spontaneously. TN pain is limited to the distribution of one or more of the divisions of the trigeminal nerve, with the maxillary division being involved in 17% of cases, the mandibular in 15%, and both branches in 32%. TN never spreads across the midline nor does it ever present on both sides of the face simultaneously.[1–3]

The peak incidence of TN occurs in the fifth to seventh decades, with 90% of cases starting after the age of 40. It is almost never seen before the third decade unless it is associated with multiple sclerosis.[4] According to Katusic *et al*. the annual incidence of TN is 4.3/100,000 and the prevalence is 15.5/100,000. There is no significant sex difference, the female/male ratio being 1.5/1. The familial type of TN presents earlier in successive generations and behaves like a disease with autosomal dominant inheritance with variable penetrance.[5]

It is suggested that unrelieved vascular compression of the trigeminal nerve entry zone initiates a focal demyelination that causes firing in the trigeminal primary afferents, which is enhanced by impairment of the inhibitory systems in the trigeminal brainstem complex.[6,7] It has been stated that increased firing of wide dynamic range (WDR) neurons in the nucleus caudalis and hypersensitivity of low-threshold mechanoreceptors (LTMs) in the nucleus oralis are responsible for the paroxysmal pain of trigeminal neuralgia, which may occur in response to noxious and non-noxious stimuli.[8]

This prospective study aimed to assess the long-term outcome of microvascular decompression (MVD) surgery and to compare it with pharmacotherapy in patients with idiopathic trigeminal neuralgia.

## Materials and Methods

This prospective study, a collaborative effort by Mansoura University Hospital in Egypt and Sebea Military Hospital in Libya, was conducted from June 2005 to September 2008 and enrolled 36 patients with a history of intractable idiopathic trigeminal neuralgia. The patients were divided into two groups: Group 1 included 21 patients who underwent MVD with 2 years' follow-up; group 2 included 15 patients who received drugs and refused MVD surgery.

A detailed history was taken from all subjects, after which clinical examination and routine laboratory tests were done; the investigations included complete blood count, blood urea, serum creatinine, fasting blood sugar, serum potassium, serum sodium, serum calcium, serum phosphorous, liver function tests, ESR, and antinuclear antibody test [Table 1].

**Table 1:** Inclusion and exclusion criteria for MVD patients

Age between 40 and 70 years Unilateral facial pain due to idiopathic TN, with no other neurological disorders Disease duration less than 5 years Only those patients who had shown poor response despite treatment with different drugs for at least 2 years prior to the surgery were included Patients with a history of a dull, aching, continuous interictal pain were excluded Patients with multiple risk factors, who were not fit for surgery, were excluded Patients who refused surgery were also excluded

Magnetic resonance imaging (MRI) of the whole brain, with particular attention paid to the region of the pons, was done for all subjects in both groups to rule out any mass lesion that could explain the occurrence of the pain. An MRI of the brain was also done in the first postoperative week for group 1 patients to exclude any complications.

All patients were followed up after surgery in the outpatient clinic for 2 years using a 10-cm visual analog scale (VAS) and the Barrow Neurological Institute (BNI) scoring system. The presence of residual pain and postoperative complications were documented. Symptom relief was assessed by comparison with the preoperative symptoms.

### Evaluation scales

We used the 10-cm VAS and the BNI scoring system for evaluating pre- and postoperative pain in all patients.[9–11]

Using the 10-cm VAS we classified response to treatment as follows[10]:

- ‘Excellent’ - if the patient indicated no pain
- ‘Failure’ - if the patient indicated the worst pain
- ‘Good’ - if the patient indicated improvement in pain
- The BNI scoring system was used to label the degree of pain relief as follows:
- Grade I - no pain and no medication required
- Grade II - occasional pain and no medication required
- Grade IIIa - no pain and continued use of medications required
- Grade IIIb - some pain, which was adequately controlled on medication
- Grade IV - pain improved but not adequately controlled on medication
- Grade V - no pain relief whatsoever

If the patient had grade IV or V symptoms after surgery they were labeled as showing ‘poor response,’ those who had grade III pain relief were considered as showing ‘good response,’ and those who had grade I pain relief were considered as showing ‘excellent response.’[11]

On the basis of both scores, the clinical outcome was plotted as ‘excellent’ if there was total absence of symptoms, as ‘good’ if some improvement of the symptoms was observed, and as ‘failure’ if the preoperative clinical status was unaffected or had become worse.

### Surgical procedure

The surgical approaches were individualized for every patient according to the anatomic and pathological interactions identified on preoperative imaging. After induction of general anesthesia, the patient was positioned in the prone, lateral decubitus, sitting, or supine position, depending on the surgeons' preference. Then a retromastoid subocciptal craniotomy was performed. The trigeminal nerve was examined microsurgically for vascular compression at or near its brainstem entry zone. Complete decompression of the trigeminal nerve was achieved using Teflon, autologous muscle tissue, or a tentorial sling.[12–15]

### Statistical analysis

The demographic, clinical, and technical data were collected using a ‘data collection form’ and entered into a computerized database before statistical analysis. Continuous variables were compared using analysis of variance for repeated measures. *P*-value < 0.05 was considered statistically significant. All data were expressed as mean ± standard deviation (SD) or patients number (n) and percentage (%) as appropriate.

## Results

The 36 patients enrolled in this study were divided into two groups: group 1 included 21 subjects (13 males and 8 females) with a mean age of 50.9 ± 7.75 years (range 40-70 years); group 2 included 15 patients (9 males and 6 females) with a mean age of 53.3 ± 8.87 years [Table 2].

**Table 2:** Demographic and clinical data of subjects in the two groups

Group	n	Age	Sex	DD	Right TN	Trigeminal nerve division n (%)				
						
		(year)	M/F	(year)	(%)	V1	V2	V3	V2+3	V1−3
1	21	50.9 ± 7.75	13/8	3.6 ± 0.87	71.4%	1 (4.8)	5 (23.8)	7 (33.3)	6 (28.6)	2 (9.5)
2	15	53.3 ± 8.87	9/6	3.23 ± 1.15	66.6%	0	3 (20)	6 (40)	5 (33.3)	1 (6.7)

V1= Ophthalmic division, V2 = maxillary division, V3 = mandibular division, M = male, F = female, n = number, DD = disease duration.

The pain scoring systems that we used identified ‘severe pain’ in 90.5% of the patients in this study. Subjects in both groups reported that the pain caused marked limitation in their daily activities and that they were dissatisfied because of the side effects associated with drug therapy.

In 18 patients, the pain disappeared on the first postoperative day; the rest of the patients reported gradual reduction of pain within 4 days of surgery. There was no recurrence throughout the 2-year follow-up period, except in one patient in whom no definite aberrant vessels were identified. This patient developed mild to moderate recurrent facial pain 1 year after surgery but the symptoms improved with a low dose of carbamazepine (400 mg per day).

Although the TN pain completely disappeared in all patients, six of them developed facial numbness after surgery; treatment was not required in any of the cases. Our final surgical results show that 17 patients had ‘excellent’ response and four had ‘good’ response; no ‘poor’ outcome was recorded. There were no major complications following surgery, apart postoperative surgical wound infection in two patients that improved without any residual consequences after an intensive course of antibiotics.

In our series, the vessels identified during surgery as causing compression were the superior cerebellar artery in 61.9% (*n* = 13), the anterior inferior cerebellar artery (AICA) in 9.5% (*n* = 2), and the transverse pontine vein in 19.1% (*n* = 4); no definite cause was identified in 9.5% (*n* = 2) of cases [Table 3]. For decompression, Teflon was used in 17 patients, muscle in three patients, and tentorial slings in one patient.

**Table 3:** Postoperative outcome after microvascular decompression

Aberrant vessel	Patient n = 21	Primary outcome	Secondary outcome	Follow-up duration in months (mean ± SD)
Sup. cerebellar A	11	Freedom from pain	Excellent to good	28.5 ± 3.6
AICA	3	Freedom from pain	Excellent	29 ± 4.1
Trans. pon. V	2	Freedom from pain	Excellent	28 ± 3.5
Pon. trigeminal V	1	Freedom from pain	Excellent	27
No definite cause	2	Freedom from pain	Good	31.5 ± 2.9
Multiple blood vessel contacts to the nerve	2	Freedom from pain	Excellent	26 ± 2.8

*P*-value of surgical group in comparison to nonsurgical group was statistically significant (*P*<0.001), Mean postoperative hospital stay 4.8 ± 0.7 days

**Figure 1:**
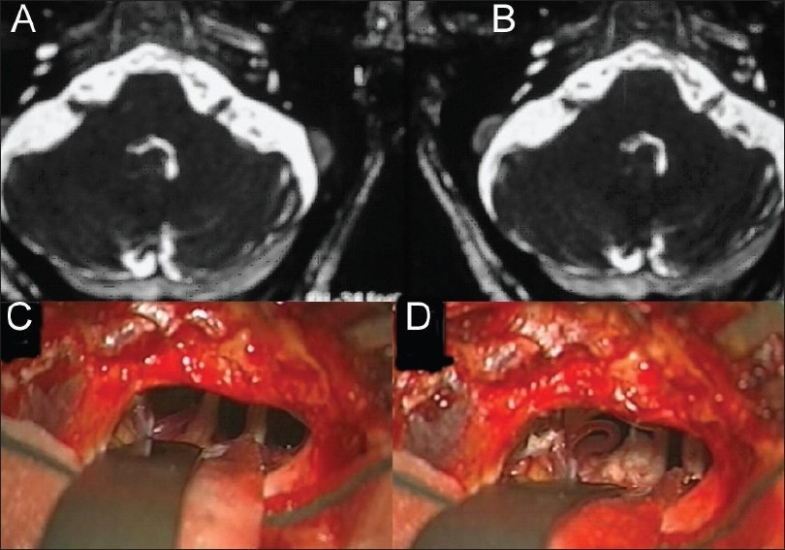
67 yo male patient has a 7 years history of right trigeminal neuralgia and no response to regular medical treatment, above image is T2 weighted MRI that showed no significant changes Apart from high suspension of right vascular loop artifacts around trigeminal nerve entry zone. A and B pictures during surgery of the same patient: picture A there is a compression of the right trigeminal nerve by superior cerebellar artery, picture B showed separation of the artery and nerve with a piece of Teflon

Preoperative MRI scans were correlated with the intraoperative findings in all patients [Figure 1]. In two out of 21 (9.5%) patients who accepted surgery in spite of no evidence of vascular compression radiologically or even intraoperatively, showed satisfactory outcomes.

Regular follow-up was done using the VAS and BNI scales every 3 months and revealed that all patients had excellent or good response postoperatively. After 12 months, all but one patient continued to report excellent response, with no recurrence of pain; the lone exception was a patient who required treatment with carbamazepine (400 mg/day) for an additional 22 months; this patient's pain was well controlled with this low dose of carbamazepine. In this study, no major complications resulted from MVD, apart from temporary hearing loss in three patients; in all of them hearing returned to normal after 4–6 months without any statistical significance hearing loss (*P* > 0.5).

In group 2, 53.3% (*n* = 8) reported poor response with pharmacotherapy over the same period of time; patients also experienced drug intolerance (*P* < 0.01) [Table 4].

## Discussion

MVD, in posterior fossa compression syndromes, has high safety and a low morbidity rate. In this study, the vascular loop compression at the root entry zone was the commonest cause of TN. Compression in this area initiates focal irritation, demyelination, and consequent devastating pain. Similar findings have also been reported by Tomasello *et al*.[16]

In nineteen (90.5%) MVD patients vessel compression of the trigeminal nerve was documented either pre- or intraoperatively. Two cases who were clinically suspected to be idiopathic TN had good response postoperatively. These results are comparable to that reported by Kabatas *et al*.[17]

On the basis anatomic studies, the transverse pontine vein is hypothesized to be the vein that is most frequently aberrant. This vein, which drains into Meckel's cave from the medial side, is usually close to the superior or inferior surface of the trigeminal nerve, just facing Meckel's cave. Missing the venous compression site might lead to a poor surgical outcome. A meticulous understanding of the venous anatomy around the trigeminal nerve and Meckel's cave may help surgeons to recognize offending veins or arteries accurately.[18–23]

In two cases, there was no definite vascular compression. This could be explained by the patients' head position, which may have separated the blood vessels from the nerve during the operative procedure. We therefore preferred to use Teflon for nerve protection.

We do not recommend surgical re-exploration in cases that do not respond satisfactorily. Such re-exploration carries a 30% risk of complications such as hearing loss and facial weakness.[18] In the current study, the sole patient who had recurrence of pain after 1 year showed excellent response to drug therapy.

In three cases, there was short-term hearing loss secondary to accumulation of fluid in the mastoid air cells (as detected by postoperative MRI); these patients regained their hearing after 4–6 months without any statistical significance residual (*P* > 0.05). The short duration of the conductive-type hearing loss was due to avoidance of cerebellar retraction during surgery. This finding is consistent with the results from other studies on MVD, which have reported death due to cerebellar hemorrhage or infarction in 1%, intracranial hemorrhage in 2%, permanent hearing loss in 3–8%, temporary hearing loss in 21%, and sensory loss in 5–31% patients.[19,20]

The current study revealed favorable outcomes that may be due to the stringent selection criteria [Table 1] that were used, the meticulous preparation, and the strict long-term follow-up. After clinical and radiological examination, those patients were excluded in whom we expected a poor postoperative outcome.

Our results [Table 3] are similar to that reported by Hamlyn and King in 1992. They stated that 90% of TN patientswho underwent MVD had vascular compression compared to just 13% of age- and gender-matched non–trigeminal neuralgia cadavers.[21]

Patients in whom MRI/MRA could demonstrate an abnormal vascular loop around the trigeminal nerve at the root entry zone usually benefitted from MVD, and this can be considered the ‘gold standard’ surgical procedure in such cases as it offers the best long-term cure rates. Outcome of MVD has also been reported to vary with the case load of the operating surgeon.[22,23]

Other therapy modalities, such as acupuncture, extracranial peripheral denervation, percutaneous radiofrequency thermocoagulation, glycerol trigeminal rhizotomy, balloon compression, and gamma knife radiosurgery, show high rates of recurrence and only short-lasting freedom from pain. These modalities are therefore suitable only for those who are medically unfit for surgery or those patients who, for some reason, have a short life expectancy.[24,25]

Matsushima *et al*. and Lee *et al*. mentioned that evidence from intraoperative documentation reveal that 80.8% of cases of compression are due to arterial compression and mostly due to the superior cerebellar artery, while 19.1% are venous compressions;[22,23] these findings are comparable to our results [Table 3].

In group 2, 46.7% of subjects had good response to drug therapy over the same period of time, though with the expected adverse effects of drug therapy [Table 4]. This is similar to the findings of Taylor *et al*. in 1981 who used carbamazepine for 16 years and found that the drug was effective in 56% of patients, though 19% experienced carbamazepine intolerance. Phenytoin's effectiveness decreases with time and less than 30% of patients continue to respond to treatment after 2 years.[26] Baclofen can be used as an adjuvant therapy, but 10% of patients cannot tolerate it. With clonazepam and valproic acid it was possible to achieve freedom from pain in 65% and 30% of cases, respectively. Pimozide is highly effective in intractable cases but it has severe adverse effects in 83% of patients and so we did not use it in this study. Topiramate, oxcarbazepine, levetiracetam, gabapentin, lamotrigine, analgesics, tricyclic antidepressants, and anxiolytics drugs had variable effectiveness as has been previously reported by Tenser and Ploghaus *et al*.[27,28]

**Table 4:** Common drugs used in this study and their adverse effects

Drug	Dose (mg/day)	Common transient adverse effects	Other possible transient adverse effects
Carbamazepine	800–1200	Nausea, ataxia, diplopia	Hyponatremia, thrombocytopenia
Oxcarbazepine	600–900	Dizziness, fatigue, headache	Rash, hyponatremia
Gabapentin	900–1600	Fatigue, lower limbs edema	Weight gain, insomnia, dizziness
Pheytoin	300–400	Lethargy, nystagmus	Rash
Amitriptyline	50–100	Somnolence, lethargy	Dry mouth, constipation
Baclofen	30–50	Drowsiness, weakness	Hypotension, constipation
Topiramate	50–150	Sedation, nervousness, paresthesia	Weight loss
Levetiracetam	500–1500	Asthenia, somnolence	Dizziness
Lamotrigine	50–150	Headache, lethargy	Nausea, tremor, insomnia
Clonazepam	2–5	Drowsiness	Ataxia

The cost of medical care for each patient was $500 a year. It was very difficult to assess the costs due to impairment of the activities of daily living and the number of working days lost. In comparison, the total cost of surgical treatment was almost $2500, with freedom from pain at least for the follow-up period in this study.

In conclusion, although TN can be managed both medically and surgically, MVD is an option that can be considered for avoiding the side effects of drugs, the adverse psychological effects of long-term pharmacotherapy, and the problems posed by comorbid conditions.

We accept that our study is underpowered. We tried to highlight the significance of early MVD in management of idiopathic TN. So, further adequately powered, multicenter trials are recommended.
